# Recent advances in optical aptasensors for biomarkers in early diagnosis and prognosis monitoring of hepatocellular carcinoma

**DOI:** 10.3389/fcell.2023.1160544

**Published:** 2023-04-18

**Authors:** Jia-Mei Dong, Rui-Qi Wang, Ning-Ning Yuan, Jia-Hao Guo, Xin-Yang Yu, Ang-Hui Peng, Jia-Yi Cai, Lei Xue, Zhi-Ling Zhou, Yi-Hao Sun, Ying-Yin Chen

**Affiliations:** ^1^ Department of Pharmacy, Zhuhai People’s Hospital, Zhuhai Hospital Affiliated with Jinan University, Jinan University, Zhuhai, Guangdong, China; ^2^ School of Traditional Chinese Medicine, Southern Medical University, Guangzhou, China; ^3^ College of Pharmacy, Jinan University, Guangzhou, China; ^4^ Guangdong Provincial Key Laboratory of Tumor Interventional Diagnosis and Treatment, Zhuhai Institute of Translational Medicine, Zhuhai People’s Hospital Affiliated with Jinan University, Jinan University, Zhuhai, Guangdong, China; ^5^ School of Stomatology, Zunyi Medical University, Zunyi, Guizhou, China

**Keywords:** optical aptasensor, hepatocellular carcinoma, biomarker, diagnosis, prognosis, monitor

## Abstract

Hepatocellular carcinoma (HCC) accounts for approximately 90% of all primary liver cancers and is one of the main malignant tumor types globally. It is essential to develop rapid, ultrasensitive, and accurate strategies for the diagnosis and surveillance of HCC. In recent years, aptasensors have attracted particular attention owing to their high sensitivity, excellent selectivity, and low production costs. Optical analysis, as a potential analytical tool, offers the advantages of a wide range of targets, rapid response, and simple instrumentation. In this review, recent progress in several types of optical aptasensors for biomarkers in early diagnosis and prognosis monitoring of HCC is summarized. Furthermore, we evaluate the strengths and limitations of these sensors and discuss the challenges and future perspectives for their use in HCC diagnosis and surveillance.

## 1 Introduction

Hepatocellular carcinoma (HCC) is the sixth most commonly diagnosed cancer and the fourth leading cause of cancer-related deaths globally (Singal et al., 2020). Owing to the lack of specific symptoms in the early stage of HCC, the diagnosis is often made at a late disease development stage, leading to a poor prognosis with an incidence-to-mortality ratio approaching one (Tsuchiya, 2015). Therefore, monitoring important HCC-related indicators has important clinical significance for their early prevention and prognostic monitoring. International guidelines broadly recommend surveillance of HCC to provide better treatment access and improvement of overall survival (Chen et al., 2020; Piñero et al., 2020).

Biomarkers refer to disease-related substances that can be detected *in vivo* or *in vitro*, such as proteins, genes, and metabolites (Liu X.-N. et al., 2019). Biomarkers can reflect the presence, development, and prognosis of the disease, serving as a basis for diagnosis, treatment, and prevention. There are several biomarkers for HCC, such as alpha-fetoprotein (AFP) (Colli et al., 2021), alpha-fetoprotein lens culinaris agglutin-3 (AFP-L3) (Choi et al., 2019), glypican-3 (GPC3) (Chen et al., 2022), sonic hedgehog ligand (SHh) (Li H.-Y. et al., 2021), and des-γ-carboxy prothrombin (DCP) (Volk et al., 2007), as well as some novel biomarkers, including osteopontin (OPN) (Cabiati et al., 2022), vascular endothelial growth factor (VEGF) (Xu et al., 2021), Golgi protein 73 (Gp-73) (Liu et al., 2022), insulin growth factor-1 (IGF-1) (Mazziotti et al., 2002), lipocalin-2 (LCN2) (Lee et al., 2015), and microRNAs (Liu et al., 2017; Chen et al., 2018). All these biomarkers are found in blood or tissue biopsies.

The sensitivity of the biomarkers varies based on the cut-off values. The cut-off values of biomarkers for HCC obtained from different clinical studies or retrospective analyses with different determination criteria are summarized in Table 1. However, the diagnostic accuracy of the absolute cut-off of these biomarkers has not been validated, and the values vary across institutions and patient populations. With a fixed tipping point of AFP (e.g., 200 ng/mL), a gradual increase rate of 7 ng/mL per month may be a useful diagnostic tool for HCC (Benson et al., 2021). The sensitivity and specificity of a single biomarker for early diagnosis of HCC vary greatly, indicating that using a single biomarker to detect early HCC is insufficient and has unsatisfactory diagnostic performance. In contrast, the combination of several biomarkers may improve early diagnostic rates.

**TABLE 1:** | Comparison of biomarkers and imaging studies in hepatocellular carcinoma.

Test items		Cut-off	Sample	Sensitivity	Specificity	Ref
AFP		400 ng/mL	serum	40%–65%	76%–96%	Benson et al. (2021), Hanif et al. (2022), Zhou et al. (2021)
AFP-L3%		10%	serum	34%	92%	Hanif et al. (2022)
GPC3		300 ng/L	serum	47.0%	93.5%	Liu (2010)
DCP		40 mAU/mL	serum	82.63%	89.12%	Ji et al. (2016)
OPN		14.64 ng/mL	serum	79.21%	63.80%	Zhu et al. (2020)
VEGF		355.2 pg/mL	serum	86.4%	60.0%	El-Houseini et al. (2005)
Gp-73		4.9 ng/mL	serum	95%	95%	Farag et al. (2019)
IGF-1		210 (185–232.5) ng/mL	serum	—	—	Elmashad et al. (2015)
LCN2		94.92 ng/mL	serum	74.3%	94.3%	Du et al. (2022)
microRNAs	miR-122	0.475	serum	81.6%	83.3%	Qi et al. (2011), Zhang et al. (2014)
	miR-143	2.21		73%	83%	
	miR-215	4.62		80%	91%	
AFP + GP73		—	serum	75%–91%	81%–97%	Hanif et al. (2022)
DCP + AFP		40 mAU/mL, 20 ng/mL	serum	91.10%	87.07%	Ji et al. (2016)
OPN + AFP		14.64 ng/mL, 20 ng/m	serum	88.12%	74.21%	Zhu et al. (2020)
AFP + AFP-L3% + DCP		20 ng/mL, 10%, 40 mAU/mL	serum	82.80%	73.2%	Lim et al. (2015)
GALAD (gender + age + AFP + AFP-L3 + DCP)		−0.63	serum	68.00%	95.00%	Best et al. (2020)
GALAD-C (gender + age + AFP + AFP-L3% + DCP)		−0.374	serum	82.60%	85.90%	Liu et al. (2020)
HES Algorithm (AFP + rate of AFP change + alanine aminotransferase + platelet count + age)		—	serum	52.56%	90%	Tayob et al. (2019)
ASAP (Age + gender + AFP + DCP)		0.5256	serum	73.8%	90.0%	Yang et al. (2019c)

AFP and liver ultrasound (US) are the most widely used methods for HCC screening. A review of serum protein biomarkers used for early detection of HCC showed that an AFP cut-off of 100 ng/mL was associated with high specificity (99%) but low sensitivity (31%). Combining AFP and US increased the sensitivity of HCC detection (97% and 78%, respectively) (Tzartzeva et al., 2018).

Abdominal multiphasic computed tomography (CT) or magnetic resonance imaging (MRI) is recommended in the setting of a rising serum AFP or following identification of a liver mass nodule ≥ 1 cm in the US, based on the guidelines of the American Association for the Study of Liver Diseases and Liver Imaging Reporting and Data System (Marrero et al., 2018), in which sensitivity for HCC is 87.5% and 83.1%, respectively (Tzartzeva et al., 2018). However, early HCC often presents as small nodules with a diameter usually less than 1 cm. CT and MRI are less sensitive in detecting small liver cancer, at 62.5% and 83.7%, respectively (Tzartzeva et al., 2018). Conversely, the detection effect of CT and MRI on HCC is susceptible to the interference of many factors such as technical level, lesions, and surrounding tissues, and false positive or false negative results may occur, leading to misdiagnosis or missed diagnosis. In contrast, biomarkers can show abnormal indications when the tumor is small or asymptomatic, detecting abnormal changes in the tumor earlier, with obvious advantages with regard to early HCC diagnosis. Consequently, clinical strategies for effectively detecting and preventing HCC metastasis recurrence are urgently required. In recent years, biosensors have provided new opportunities and have attracted great interest among researchers.

**FIGURE 1:**
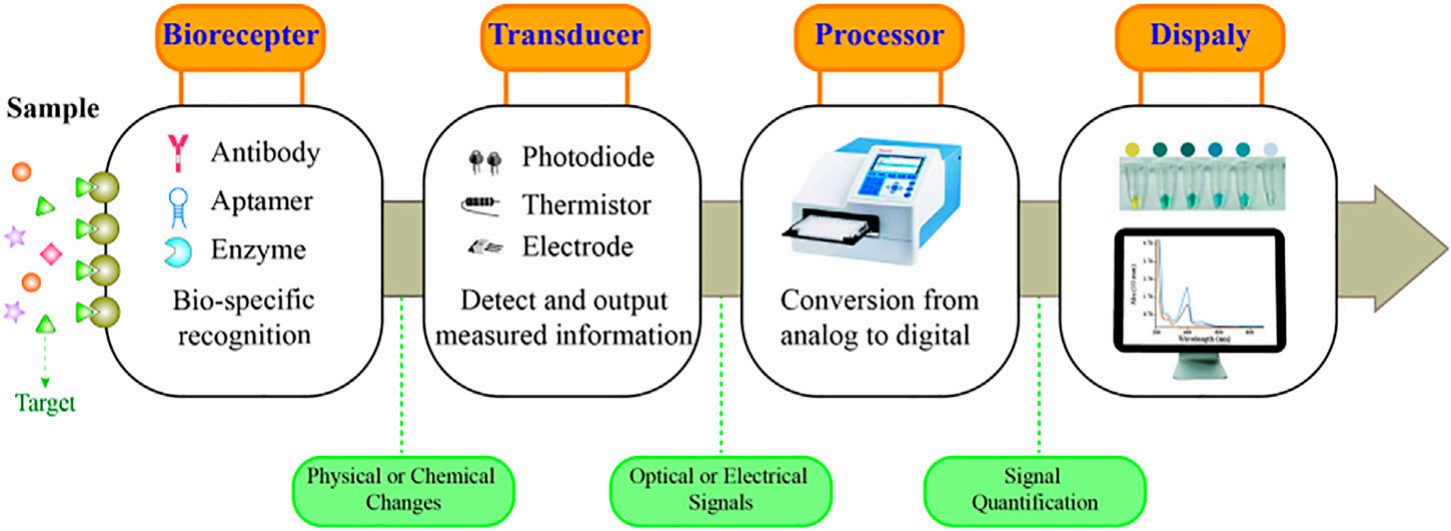
Schematic representation of a biosensor.

A typical biosensor consists of a biorecognition element and a transducer that can provide qualitative or quantitative results of the targets (Figure 1) (Lim et al., 2010). Common bioreceptors comprise antibodies, aptamers, and enzymes (Bhalla et al., 2016). Among them, aptamers are oligonucleotides, short DNA, or RNA obtained by SELEX screening (Hosseinzadeh and Mazloum-Ardakani, 2020). Aptamers offer numerous advantages over the already widely used antibodies, such as a wide range of targets, high specificity and affinity to targets, low synthetic cost, and easy chemical modification (Li W. et al., 2015; Dehghani et al., 2018; Ibau et al., 2019). Aptamer-based sensors are also referred to as aptasensors, which use aptamers as the biorecognition element. Among them, optical aptasensors are biosensors that incorporate aptamers as the biorecognition element and various optical analytical techniques as the signal transductions, integrating the advantages of the aptamer and optical analysis (Feng et al., 2014; Sadeghi et al., 2018; Zahra et al., 2021). Hence, optical aptasensors can provide potential opportunities for the detection of targets in clinical analysis.

Currently, biomarkers of HCC can be detected by various analytical methods, including enzyme-linked immunosorbent assay (ELISA) (Hanif et al., 2022), mass spectrometry (MS) (Lyman et al., 2022), fluorescence quantitative PCR (qPCR) (Yang J. et al., 2019), immunohistochemistry (IHC) (Yang J. et al., 2019), chemiluminescence (CLIA) (Sun et al., 2018), and biochip technology (Zhang et al., 2023). In contrast, the high specificity of the aptasensor is prominent such that targets as low as fg/mL levels can be detected. Furthermore, the aptasensor can complete the detection in a few minutes, which is more rapid than other detection methods. They can also monitor changes in biomarkers in real time and facilitate timely diagnosis and treatment decisions. Certainly, aptasensors have some limitations that need to be addressed. At present, most of the aptasensors can only detect a specific type of target, and it is necessary to develop aptasensors that can simultaneously detect multiple targets. In addition, although the synthesis of aptamers is relatively low, the cost of aptasensors is limited by the enzymes and nanomaterials.

In the present review, we outline the recent advancements in aptasensors based on several optical principles in HCC surveillance and diagnosis, including fluorescence, colorimetry, chemiluminescence, surface plasmon resonance (SPR), and surface-enhanced Raman scattering (SERS). We focused on the detection methods of biomarkers associated with the early diagnosis and poor prognosis of HCC. With examples of various sensors, the common optical and color rendering materials and the working principle of these aptasensors are introduced. Besides, Table 2 summarizes the developments and applications focusing on the biomarker, material, detection limit, and linear range. Furthermore, the strengths and limitations of the sensors are evaluated and summarized in Table 3. Ultimately, we discuss the challenges and opportunities encountered in the application of the sensors in the surveillance and diagnosis of HCC.

**TABLE 2:** | Summary of biomarkers related to hepatocellular carcinoma (HCC) surveillance and diagnosis using optical aptasensors.

Detection method	Marker	Material	LOD	Linear range	Ref
Fluorescence	AFP	CdTe quantum dots (QDs); anti-AFP antibody functional gold nanoparticles (AuNPs)	400 pg/mL	0.5–45 ng/mL	Zhou et al. (2019)
	AFP	molybdenum disulfide (MoS_2_) nanosheets; gold nanoclusters (Au NCs)	0.16 ng/mL	0.5–120 ng/mL	Xu et al. (2017)
	AFP	graphene oxide (GO); fluorescein amidite (FAM; cyanine dye Cy3; cyanine dye Cy5; Fluor Alexa 405)	0.45 nmol/L	0.8–160 mol/L	Xu et al. (2019)
	VEGF	GO; fluorescein dye	0.30 nmol/L	0.5–100 nmol/L	
	AFP	A polydopamine nanosphere@silver nanocluster (PDAN@AgNC) system	2.4 nmol/L	10–100 mol/L	Jiang et al. (2019)
	AFP	5-carboxyfluorescein (FAM); palladium nanoparticles (PdNPs)	1.4 ng/mL	5.0–150 ng/mL	Li et al. (2019)
	AFP	Fe_3_O_4_@AuNPs (AuMPs) magnetic beads; single-stranded DNA primers; antibodies; hairpins	0.1 pg/mL	0.001–10 ng/mL	Xie et al. (2019)
	AFP	GO; 5-carboxyfluorescein (FAM)	0.909 pg/mL	0–300 pg/mL	Wang et al. (2018a)
	AFP	mismatched catalytic hairpin assembly (MCHA); 5-carboxyfluorescein (FAM); BHQ1 (quencher)	0.033 ng/mL	0.1 ng/mL–10 mg/mL	Li et al. (2021b)
	AFP	polyfluorene-based cationic conjugated polyelectrolytes (PFN^+^)	1.76 ng/mL	10–1,000 ng/mL	Bao et al. (2018)
	AFP	GO; 5-carboxyfluorescein (FAM)	43 fmol/L	0–10 pmol/L	Zhang et al. (2016)
	AFP	N-methyl mesoporphyrin IX; QDs; two-aptamer double-stranded DNA; G-quadruplex sequence and Ag+	3 fg/mL	10–1,000 fg/mL	Chen et al. (2022)
	GPC3		0.25 fg/mL	1–1,000 fg/mL	
	GPC3	glutathione@graphene quantum dots (GSH@GQDs); reductive GO (RGO)	2.395 ng/mL	5–150 ng/mL	Wang et al. (2022a)
	SHh	microbeads, Texas-Red, and BHQ2 (quencher)	69 pmol/L	0.07–62.5 nmol/L	Li et al. (2021a)
	LCN2	3,3′,5,5′-tetramethylbenzidine (TMB); horseradish peroxidase (HRP)	0.6 ng/mL	2.5–500 ng/mL	Lee et al. (2015)
	VEGF	upconversion nanoparticles (UCNPs); molybdenum disulfide MoS_2_ nanosheets	0.1 ng/mL	0.1–16 ng/mL	Yuan et al. (2020)
	VEGF	porphyrin-based covalent organic framework (p-COF); carbon dots (CDs)	20.9 fg/mL	1.0 pg/mL–100 ng/mL	Cui et al. (2021)
	VEGF	DNA polymerase, nicking enzyme; fluorescent/quencher labeled probe	3.5 pg/mL	5–400 pg/mL	Li et al. (2017)
	VEGF	FAM (fluorophore); BHQ1 (quencher)	—	0.05–6 ng/mL	Lu et al. (2018)
	VEGF-165	poly-L-lysine-coated gold nanoparticles (AuNPs); fluorescent probe	1.25 pmol/L	1.25 pmol/L–.25 μmol/L	Cho et al. (2012)
	VEGF-165	silver nanoparticles (AgNPs); Mn-doped ZnS QDs; BHQ2 quencher-label	0.08 nmol/L	0.1–16 nmol/L	Zhu et al. (2015)
	VEGF-165	GO; nicking enzyme	1 pmol/L	1 pmol/L–4 nmol/L	Li et al. (2015b)
Colorimetric	OPN	AuNPs; nitrocellulose membrane	0.1 ng/mL	10–500 ng/mL	Mukama et al. (2020)
	OPN	cellulose paper chemically modified with (mercaptopropyl)methyl-dimetoxisilane; Bradford reagent	5 ng/mL	5–1,000 ng/mL	Pereira et al. (2022)
	VEGF	AuNPs; DNA dendritic nanostructure	185 pmol/L	185 pmol/L–7.4 nmol/L	Chang et al. (2016)
	VEGF	AuNPs	0.1 nmol/L	0.1–40 nmol/L	Wu et al. (2016)
	VEGF	G-quadruplex DNAzyme; ABTS -H_2_O_2_ system	1.7 pmol/L	24 pmol/L–11.25 nmol/L	Zhang et al. (2017)
	VEGF-165	streptavidin labeled-horseradish peroxidase (HRP-SA); TMB; H_2_O_2_ system	10 pg/mL	0.1–100 ng/mL	Dong et al. (2020)
Chemiluminescence (CL)	AFP	hemin@ZIF-67 composites; luminol-H_2_O_2_-NaOH	0.13 ng/mL	0.4–20 ng/mL	Wang et al. (2022b)
	AFP	restriction enzyme digestion; gold nanoparticles; horseradish peroxidase (HRP)	0.94 ng/mL	2–500 ng/mL	Yan et al. (2022)
	AFP	metal-organic frameworks (Fe-MOFs); H_2_O_2_; luminol	77 pg/L	0.1 ng/L–300 mg/L	Han et al. (2020a)
	VEGF	guanine; 5-carboxy fluorescein (FAM); paramagnetic beads	0.4 ng/mL	0.8–100 ng/mL	Chen et al. (2020)
	VEGF-165	silica (SiO_2_); horseradish peroxidase (HRP)	2.15 pmol/L	—	Pasquardini et al. (2015)
	VEGF-165	CdSe/ZnS QDs-hemin/G-quadruplex supramolecular structure; black hole quencher pair and Exonuclease III	5 pmol/L	10 pmol/L–100 nmol/L	Freeman et al. (2012)
	VEGF-165	manganese porphyrin probe (MnPyP) probe	50 pmol/L	0–25 nmol/L	Li et al. (2015a)
	VEGF-165	4-methoxy-4-(3-phosphatephenyl)-spiro-(1,2-dioxetane-3,2-adamantane) (AMPPD); alkaline phosphatase (ALP) and streptavidin-coated magnetic beads (MBs-SA)	1 ng/mL	1–20 ng/mL	Shan et al. (2017)
Surface-enhanced Raman scattering (SERS)	AFP	DNA hydrogel; immunoglobulin G (IgG)	50 pg/mL	50 pg/mL–0.5 μg/mL	Wang et al. (2020)
	AFP	gold-silver-silver core shell-shell nanotrepangs (GSSNTs); MBs-SA	35 particles/μL	0–107 particles/μL	Ning et al. (2020)
	AFP	AgNPs; ssDNA (DNA1,DNA2)	0.097 amol/L	0.2–20 amol/L	Wu et al. (2015)
	VEGF	Ag–Au Pys nanostructures (Ag–Au Pys)	22.6 amol/L	0.01–1.0 fmol/L	Zhao et al. (2015)
	VEGF	Au nanoparticles modified magnetic Fe_3_O_4_ nanoparticles (Fe_3_O_4_/AuNPs)	2.3 pg/mL	0.01–50.0 ng/mL	Huang et al. (2022)
Surface plasmon resonance (SPR)	VEGF	Carboxyl-coated polystyrene microspheres (PSMs); phi29 polymerase; dNTPs	100 pg/mL	100 pg/mL −1 μg/mL	Chen et al. (2014)
	VEGF	plastic optical fiber (POF); gold fiber film	0.8 nmol/L	—	Cennamo et al. (2015)
	miRNA-21	AuNPs; AgNPs	0.6 fmol/L	—	Liu et al. (2017)
Electrochemiluminescence (ECL)	AFP	silica nanoparticles doped thionine (Th@SiO_2_ NPs); Ru (bpy)_3_^2+^/tripropylamine (TPA)	—	7 0 pg/mL–0.14 ng/mL	Wu et al. (2015)
	VEGF-165	T7 exonuclease (T7 Exo); G-quadruplex/hemin DNAzyme; CdS: Eu nanocrystals (CdS: Eu NCs)	0.2 pmol/L	1 pmol/L–20 nmol/L	Zhang et al. (2015)
Resonance light scattering (RLS)	AFP	double-stranded DNA	0.94 μg/L	5–100 μg/L	Chen et al. (2018)
	miRNA-122		98 pmol/L	200 pmol/L–10 nmol/L	
Liquid crystals (LCs)	AFP	glass slide; dimethyloctadecyl (3-(trimethoxy-silyl)propyl)ammonium chloride (DMOAP); 3-Aminopropyltriethoxysilane (APTES); glutaraldehyde (GA)	12.62 pg/mL	20–800 pg/mL	Duong and Jang (2021)
	AFP	magnetic beads (MBs); signal DNA; cationic surfactant	0.19 ng/mL	0.46–3 ng/mL	Qi et al. (2020)

**TABLE 3:** | Summary and comparison of different aptasensor platforms.

Optical analysis strategy	Characteristics	Advantages	Challenges
Fluorescence	a large number of targets, whether in solution or on the membrane	high sensitivity, convenience	photobleaching can affect long-term usability
Colorimetric	simple sensing mode and signals conversion from invisible signals to megascopic color changes	visible radiation, easy operation, rapid reading, low-cost	high detection limits compared to other methods
Chemiluminescence (CL)	wide calibration ranges and simple instrumentation	high sensitivity, good accuracy, and precision	limited to certain targets and sample types
Surface plasmon resonance (SPR)	suitable in order that measuring local refractive index changes arising from the adsorption of targets	easy operation and high sensitivity	expensive instrumentation and labels involving enzyme substrates and fluorescent materials
Surface-enhanced Raman scattering (SERS)	multiplexing capability	non-destructive and label-free detection method	signal enhancement requires careful optimization
Electrochemiluminescence (ECL)	exceptional photophysical properties	high sensitivity and specificity and wide dynamic range	electrochemical interference and background noise
Resonance light scattering (RS)	different probes and receptors for sensing	high sensitivity and selectivity can detect trace or ultra-trace samples with no need for labeling or complex instruments	limited applications for certain targets and sample types

## 2 Optical aptasensors

### 2.1 Fluorescence aptasensors

The light emitted by the normal temperature material following the absorption of electromagnetic energy is called fluorescence. Fluorescence-based strategies are often employed in constructing aptasensors for the target molecules due to advantages such as high sensitivity, strong specificity, and simple operation (Jia et al., 2020). Förster resonance energy transfer (FRET) occurs where dipole-mediated energy transfer occurs from the excited donor fluorophore to a proximal ground-state receptor (Kaur et al., 2021). In the design of fluorescent aptasensors, FRET has been used extensively as the significant sensing form based on fluorescent quenching or fluorophore signal recovery strategies (Cao et al., 2012; Shan et al., 2014; Yang B. et al., 2018).

**SCHEME 1:**
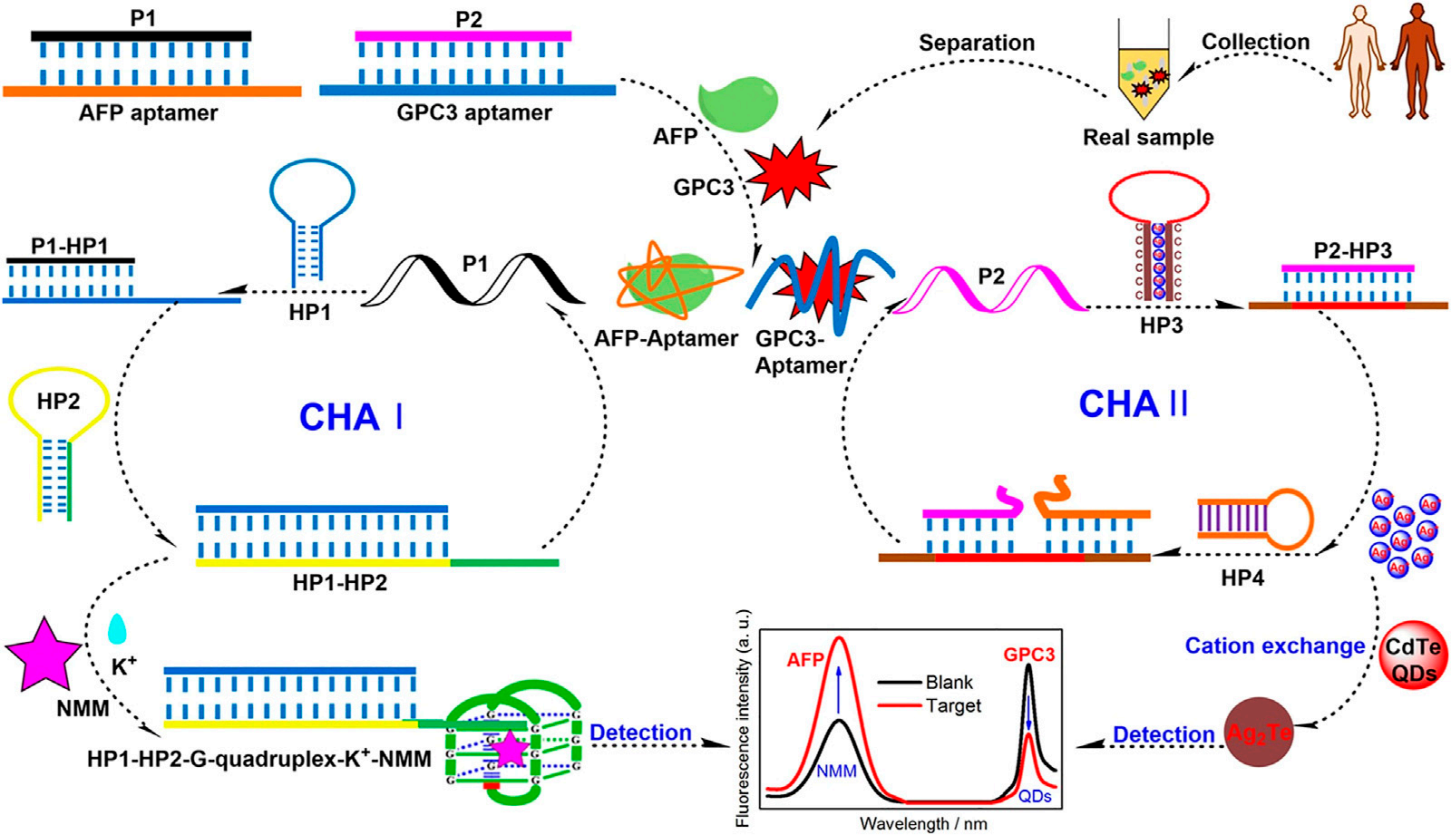
Schematic illustration of a Förster Resonance Energy Transfer (FRET) aptasensor for simultaneous detection of alpha-fetoprotein (AFP) and Glypican-3 (GPC3) (Chen et al., 2022). Reprinted (adapted) with permission from Chen et al. (2022). Copyright © 2022 American Chemical Society.

Over the past few decades, a series of FRET biosensors have been designed to detect diverse tumor markers with organic dyes or semiconductor quantum dots (QDs) as energy donors (Wang et al., 2014). Wang H. et al. (2022) labeled the GPC3 aptamer with glutathione@graphene QDs to construct the GSH@GQDs-GPC3Apt complex. Based on the complex, they designed a novel FRET biosensing platform for GPC3 detection, with a linear range from 5 ng/mL to 150 ng/mL and a limit of detection of 2.395 ng/mL at an S/N of three. Chen et al. reported a strategy for the simultaneous detection of AFP and glypican-3 GPC3 by developing a catalytic hairpin assembly amplification method with Nmethyl mesoporphyrin IX and QDs as signal reporters. The limits of detection of the assay were as low as 3 fg/mL for AFP and 0.25 fg/mL for GPC3 (Scheme 1) (Chen et al., 2022).

Li H.-Y. et al. (2021) devised a simple aptasensor for detecting SHh content by conjugating Texas-Red-labeled aptamer for SHh to microbeads. The broad detection range was from 0.07 to 62.5 nM, and the LOD was down to 69 pM. In addition, the test results of 28 HCC specimens demonstrated that the method compensated for detecting HCC in AFP-negative cases (Li H.-Y. et al., 2021).

**SCHEME 2:**
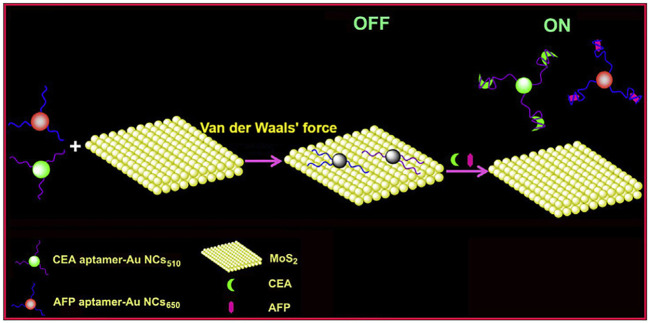
A Förster Resonance Energy Transfer (FRET) biosensor for simultaneous detection of multiple tumor markers (Xu et al., 2017).

The effective application of organic dyes and QDs is limited due to the easy photo-bleaching, poor photo-stability of organic dyes, and high toxicity for QDs. In comparison, gold nanoclusters (AuNCs) have become the new candidates owing to their great biocompatibility, stable fluorescence emission, and good photo-stability (Yang et al., 2014; Ding and Tian, 2015). Based on this, Xu et al. (2017) described a novel aptamer-induced “switch on” FRET aptasensor for the simultaneous determination of multiple tumor target markers (e.g., AFP) by virtue of combining MoS_2_ nanosheets and multicolored AuNCs through single wavelength excitation (Scheme 2). The FRET biosensor used AFP aptamer functionalized dual-colored gold nanoclusters (Au NCs) as energy donors and MoS_2_ as energy receptors. This method demonstrated that the detection limit decreased to 0.16 ng/mL at 3 s, with a linear range of 0.5–120 ng/mL for AFP. Moreover, the biosensing platform can visually and qualitatively discriminate serums from normal and hepatoma patients, demonstrating the assay potential for clinical diagnosis (Xu et al., 2017).

### 2.2 Colorimetric aptasensors

Colorimetric aptasensors have been used to detect tumor biomarkers due to their ease of use, accessibility, and point-of-care detection (Wang D. et al., 2018). Colorimetric biosensing platforms are a promising technique as they translate invisible signals into visual color changes. The test results are directly assessed by the naked eye without the support of any instrument; hence, this type of sensor has the greatest commercial testing potential (Xiao et al., 2017; Akshaya et al., 2020). The most common catalysts in the colorimetric methods are horseradish peroxidase (HRP) and an HRP-mimicking DNAzyme named G-quadruplex-hemin DNAzyme for reactions between the chromogenic substrate and hydrogen peroxide (H_2_O_2_). These catalysts can effectively catalyze H_2_O_2_-mediated oxidation of 2,2-azino-bis (3-ethylbenzothiazoline-6-sulfonicacid), diammonium salt, and 3,3′,5,5′-tetramethylbenzidine (TMB), with a visible colorimetric output signal (Yang B. et al., 2019).

**SCHEME 3:**
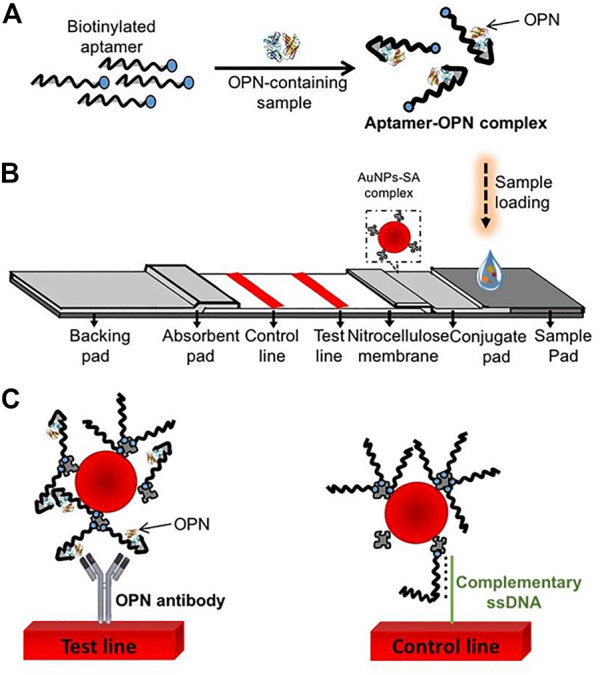
Scheme of the osteopontin (OPN) lateral flow biosensor (LFB). **(A)** Capture of the targets by the aptamers; **(B)** The LFB assembly with streptavidin-coated gold nanoparticles (AuNPs-SA) on the conjugate pad, immobilized OPN antibody, and a probe complementary to the aptamer on the nitrocellulose membrane; **(C)** the anti-OPN antibody at the test line captured the OPN-aptamer, and the spare aptamers are captured by the complementary ssDNA probes at the control line (Mukama et al., 2020).

The lateral flow biosensor (LFB) is an ideal platform for the rapid detection of various cancer biomarkers, integrating four major parts: samples, conjugates, absorption pads, and the nitrocellulose membrane (Sajid et al., 2015; Baryeh et al., 2017; Huang et al., 2017; Mukama et al., 2017). Mukama et al. established a simple, rapid, and highly specific LFB for OPN detection (Scheme 3). This is an example of collaboration between the aptamer and antibody. The aptamer for OPN was used for pre-capture from samples, and the antibody for OPN was immobilized on the test line for a second identification. Finally, gold nanoparticles (AuNPs) sprayed on the conjugation pad were utilized for color detection. Notably, the LFB achieved a qualitative and semi-quantitative detection of OPN in clinical serum and could provide a rapid visual detection response within 5 min (Mukama et al., 2020).

**SCHEME 4:**
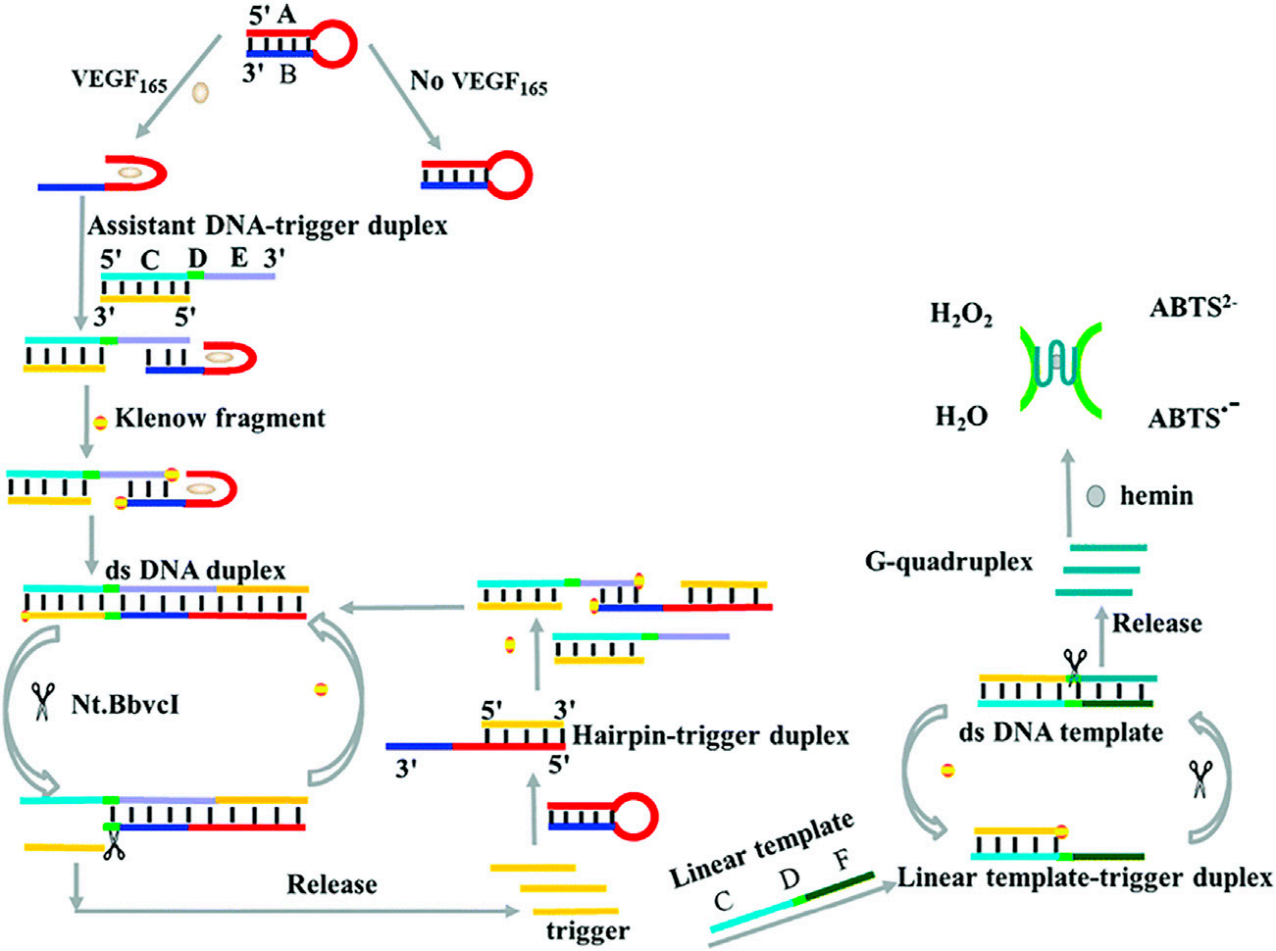
Illustration of VEGF-165 detection based on a colorimetric biosensor (Zhang et al., 2017).

To improve the sensitivity of the colorimetric biosensor based on G-quadruplex-hemin DNAzyme, Zhang et al., 2017 introduced strand displacement amplification (SDA) as an amplification technique for developing a label-free colorimetric aptasensor for sensitive detection of VEGF-165 (Scheme 4). The specific binding of VEGF-165 with the aptamer-based hairpin probe initiated the SDA to produce more triggers, which can further anneal with the hairpin probe and result in the opening of the hairpin structure, subsequently initiating the next round of SDA reaction and yielding more triggers. Finally, the abundant triggers hybridize with the linear template and subsequently initiate a new round of SDA reactions to generate additional G-quadruplex-hemin DNAzymes for further colorimetric reactions. The linear relation ranges from 24 pM to 11.25 nM, with a detection limit of 1.7 pM (Zhang et al., 2017). In addition, Dong et al. (2020) designed a high-sensitivity colorimetric aptasensor using HRP-catalyzed TMB and hydrogen peroxide systems as outcome signals for VEGF-165 detection in human serum. The strategy achieved a low detection limit of 10 pg/mL and had a broad linear range of 0.1–100 ng/mL (Dong et al., 2020).

### 2.3 Chemiluminescence aptasensors

A phenomenon where the light emitted by a substance does not result from heat is called luminescence, including chemiluminescence (CL) and electrochemiluminescence (ECL). CL is a photoradiation phenomenon that occurs during a chemical reaction. C-based strategies are widely applied in developing aptasensors employed in cancer biomarker detection owing to their relatively high sensitivity, wide calibration ranges, and repeatability (Eivazzadeh-Keihan et al., 2017; Kou et al., 2020). The monitoring of VEGF based on CL development has been studied extensively.

**SCHEME 5:**
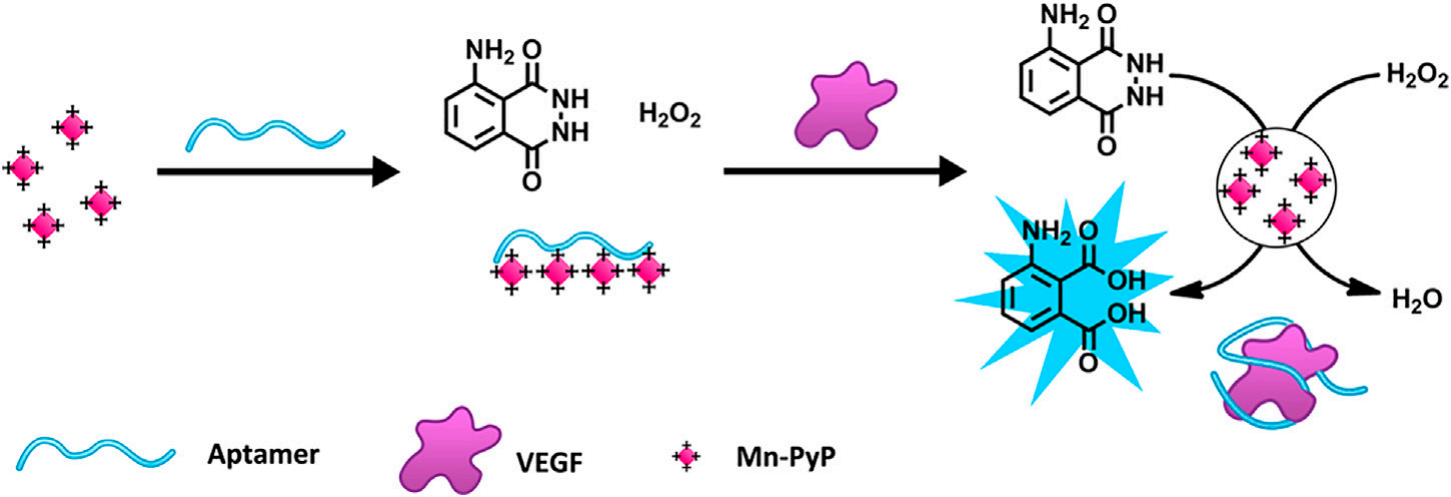
Schematic Illustration of the porphyrin probe (Mn-PyP) based on a vascular endothelial growth factor (VEGF) sensing strategy (Li W. et al., 2015). Reprinted (adapted) with permission from Li W. et al. (2015). Copyright 2015 © American Chemical Society.

The luminol reaction with H_2_O_2_ is one of the most commonly used and reliable CL reactions (Khan et al., 2014). Li W. et al. (2015) designed a chemiluminescence aptasensor based on the aptamer-controlled non-covalent porphyrin probe self-assembly for VEGF detection (Scheme 5). A positively charged porphyrin probe (Mn-PyP) as a catalyst could catalyze the luminol CL reaction. Without VEGF, the Mn-PyP catalyzed CL reaction is efficiently suppressed because of the VEGF aptamer-induced aggregation of Mn-PyP. Therefore, the target turns on the CL reaction because of the high affinity between VEGF and the VEGF aptamer. The detection limit of the method for VEGF is 50 pM, and the concentration ranges from 0 to 25 nM (Li W. et al., 2015). Wang J. et al. (2022) detected AFP based on the CL system of hemin@ZIF-67–H_2_O_2_-NaOH, achieving a detection limit of 0.13 ng/mL. In a study conducted by Han R. et al. (2020), a sensing platform based on the luminol–H_2_O_2_–metal organic framework (MOF) system was constructed for AFP detection. The aptasensor had an LOD of 77 pg/L for AFP (Han R. et al., 2020).

**SCHEME 6:**
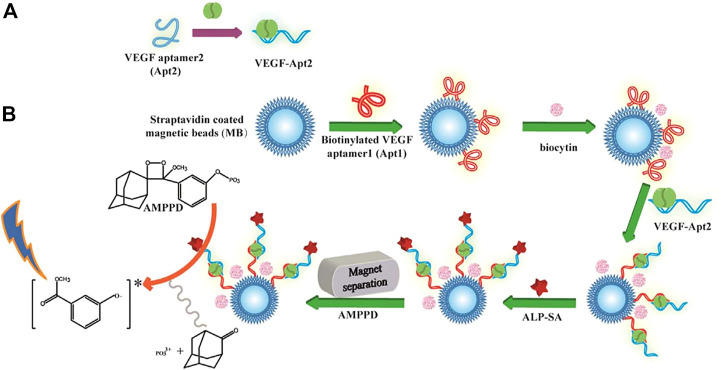
Schematic representation of the chemiluminescence aptasensor for the quantitative determination of VEGF-165 (Shan et al., 2017). **(A)** VEGF165 sample was incubated with Apt2 solution. **(B)** Apt1 immobilization on MBs and coupling with VEGF-Apt2 for following ALP-AMPPD CL detection.

Another common reaction used for CL is the hydrolysis of 4-methoxy-4-(3-phosphatephenyl)-spiro-(1,2-dioxetane-3,2-adamantane; AMPPD) under the catalysis of alkaline phosphatase (ALP) (Li et al., 2010; Yue and Liu, 2013; Fan et al., 2021). Shan et al. (2017) developed a sensitive and selective CL method for quantitative VEGF-165 detection (Scheme 6). Considering the two binding domains in VEGF-165, the researchers designed a dual-aptamer-based sandwich structure, where one capture aptamer was immobilized on magnetic beads (MBs), and another aptamer was labeled by biotin for further ALP conjunction. After the target-induced Apt-VEGF-Apt sandwich formed on the surface of the MBs, ALP would bind to the second aptamer by primary magnetic separation to remove excess Apt-biotin. Subsequently, after secondary magnetic separation, ALP catalyzed AMPPD, and a strong chemiluminescence signal was produced. Notably, the method has been successfully applied to a hypoxic co-culture model with excellent accuracy, equal to that of an ELISA Kit (Shan et al., 2017).

### 2.4 Surface-enhanced Raman scattering-based aptasensors

The underlying principle of surface-enhanced Raman scattering (SERS) is that the Raman scattering intensity after molecular adsorption on the metal surface is improved, including electromagnetic enhancement (EM) and chemical enhancement (CM) (Ding et al., 2017; Mohammadpour et al., 2017; Joseph et al., 2018). Precious metal nanoparticles (i.e., Au or Ag NPs) are often used as probes to label SERS due to their plasmonic properties (Mosier-Boss, 2017). SERS has been applied extensively in biomedical research, clinical diagnosis, and food safety control (Jiang et al., 2015; Bryche et al., 2017).

**SCHEME 7:**
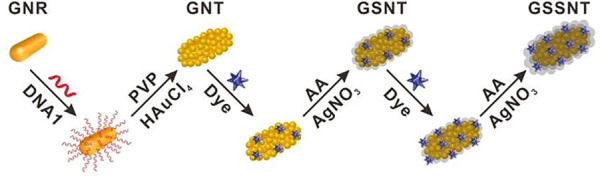
Schematic illustration of the fabrication of Surface-enhanced Raman scattering (SERS)-active nanotags (Ning et al., 2020).

Ning et al. (2020) developed a SERS-based aptasensor for the simultaneous sensitive detection of multiple cancer-related exosomes, including AFP (Scheme 7). They synthesized multiplexed capture probes composed of bumpy surface nanorod (gold nanotrepang, GNT) cores and bilayer silver shells. The three types of gold-silver-silver core shell-shell nanotrepangs (GSSNTs) were decorated on the MBs via the functionalization of linker DNAs, which were complementary to the aptamers for the targets. In the absence of the target, SERS detection probes were coupled with MBs via specific DNA hybridization as an aptamer-based SERS sensor. In the presence of the target, the aptamer specifically captured the target, and GSSNTs were subsequently released into the supernatant resulting in attenuated SERS signals. The quantitative detection of every single target and multiplex assays renders this sensor highly promising in clinical diagnosis (Ning et al., 2020).

**SCHEME 8:**
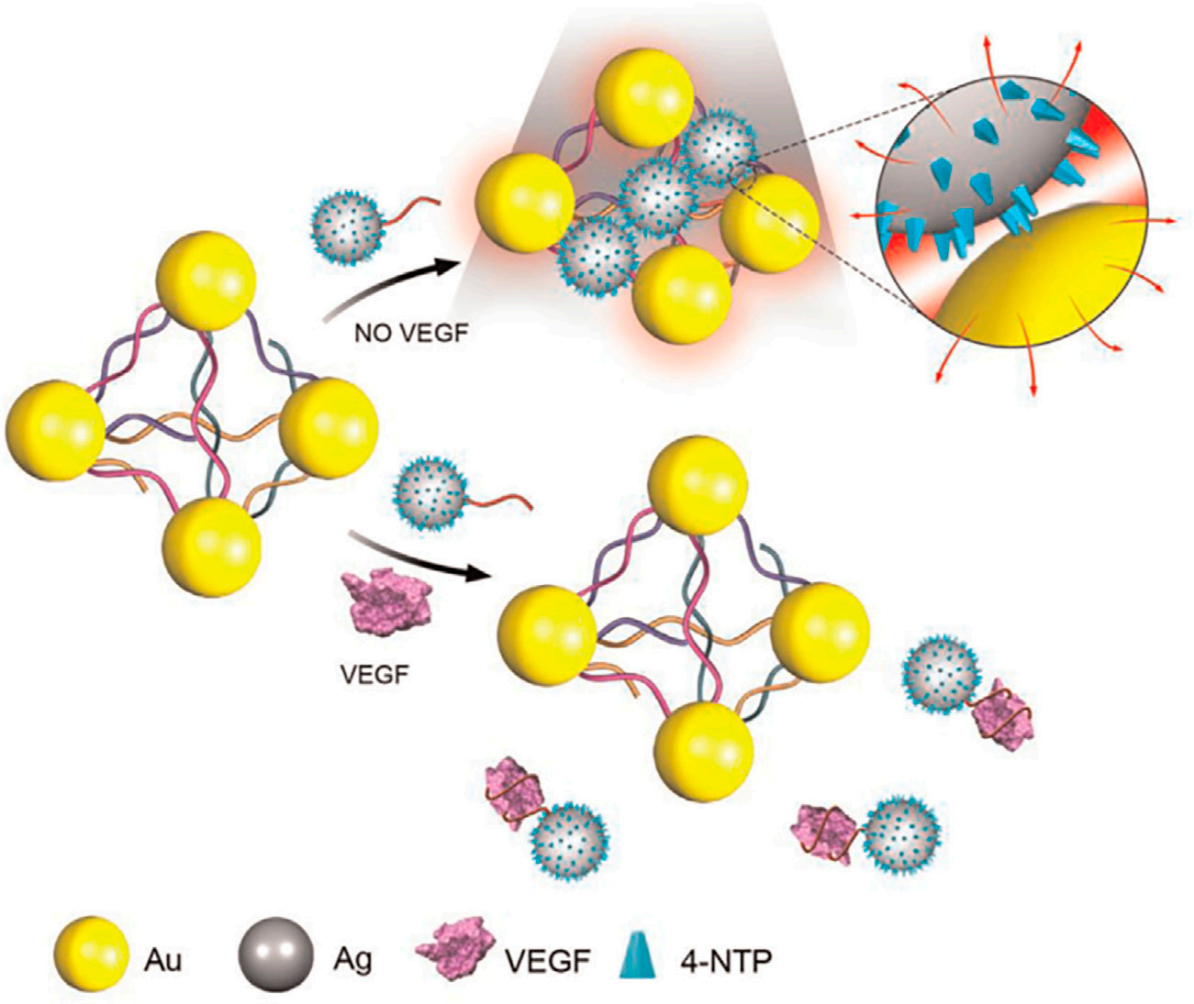
Schematic illustration for Surface-enhanced Raman scattering (SERS) assay detection of Vascular Endothelial Growth Factor (VEGF) based on self-assembled Ag ornamented–Au pyramid superstructure (Zhao et al., 2015).

Zhao et al. (2015) demonstrated the fabrication of a novel 6-nm Ag ornamented–10 nm Au nanoparticle pyramid-like superstructure (Ag–Au Pys), in which the number of hotspots between silver nanoparticles (AgNPs) and AuNPs had a good relationship with the SERS intensity (Scheme 8). The investigators used the aptamer for VEGF and its partially complementary sequence to assemble Ag–Au Pys nanostructures under optimized conditions. The detection limits of VEGF decreased to 22.6 aM within a wide linear range of the VEGF concentration of approximately 0.01–1.0 fM (Zhao et al., 2015).

### 2.5 Surface plasmon resonance-based aptasensors

Surface plasmon resonance (SPR) is a sensitive surface analysis technology that detects changes in the refractive index when molecular adsorption occurs on heavy metal membranes (Nguyen et al., 2015). SPR has been used widely for studies of biomolecular interactions since the 1990s. However, routine SPR testing cannot detect small changes in refractive coefficient, limiting its application in hypersensitivity detection. Therefore, SPR techniques have been developed with amplification methods.

**SCHEME 9:**
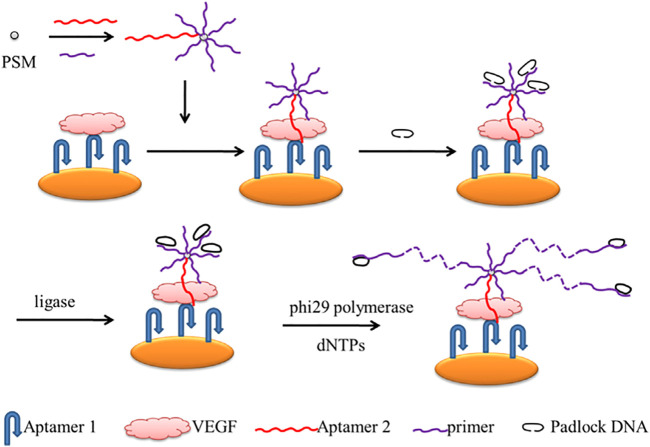
Scheme of the Rolling Circle Amplification (RCA)-assisted Surface Plasmon Resonance (SPR) sensing platform (Chen et al., 2014).

Chen and his team described a rolling circle amplification (RCA)-assisted SPR sensor for detecting VEGF (Scheme 9). In the strategy, two aptamers and the primer that possessed the complementary sequence of the padlock probes were used to initiate the RCA reactions. The 30-NH_2_ Aptamer2 and 30-NH_2_ primer were loaded on carboxyl-coated polystyrene microspheres through amidation. Aptamer1 was immobilized on the gold surface to bind VEGF. To obtain a well-aligned Aptamer1, monolayers of mercaptohexanol were modified to fill the gold chip to prevent non-specific binding. When the target activated the sensor, it indirectly triggered the RCA reactions. With the help of ligase, the RCA is performed under the phi29 polymerase and dNTPs. Therefore, VEGF can be monitored very sensitively and selectively using this RCA-assisted SPR sensor. With the sensitive and selective platform, VEGF can be monitored in a linear range of 100 pg/mL–1 μg/mL, with a detection limit of 100 pg/mL (Chen et al., 2014).

**SCHEME 10:**
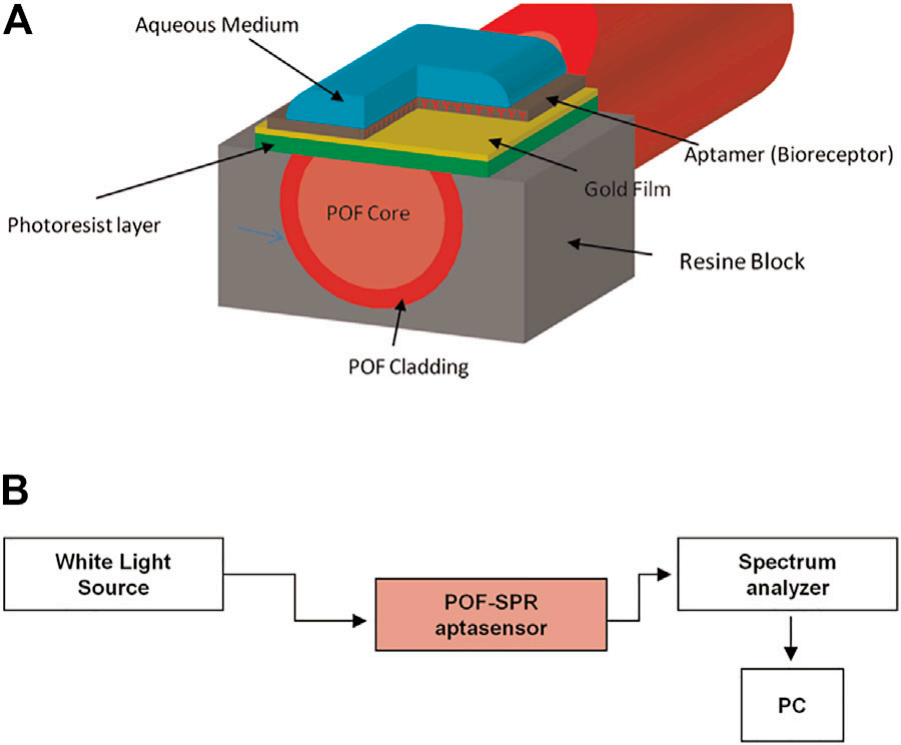
**(A)** Schematic illustration of the Plastic Optical Fiber-Surface Plasmon Resonance (POF-SPR) aptasensor, with a cross-section of the sensor system. **(B)** Experimental setup (Cennamo et al., 2015).

Based on the SPR mechanism, Cennamo et al. developed a rapid, portable, and low-cost diagnostic platform taking advantage of a plastic optical fiber (POF) to detect VEGF (Scheme 10). To achieve good sensitivity and signal-to-noise ratio, the preparation process is complex. In brief, the POF was first designed as a PMMA core of 980 μm with a fluorinated polymer cladding of 20 μm. In addition, the coating of the POF was removed along half of the circumference, and the Micropotential S1813 photoresist buffer was rotated on the exposed core and finally sputtered with a thin gold fiber film using a sputtering machine. Researchers also compared two different types of modification on the surface of the gold fiber film; one immobilized with aptamer only (Apt) and one with aptamer and passivating agent (mercaptoethanol) (Apt-MPET). The results indicated that a better interface was fabricated when the passivation molecule was fixed on the sensor surface after the aptamer (Apt-MPET). The sensitivity was about 8.3 times superior to that of the non-passivated sensor, and the detection limits were 0.8 nM and 3 nM, respectively. However, the passivation process needs to be optimized further to enhance the affinity and kinetic properties of the aptamer. For example, using the method mentioned by Cennamo et al., adding a polyethylene glycol (PEG) layer between the gold fiber layer and the aptamer layer can reduce non-specific adsorption and result in a partial loss of molecule affinity for the target. The underlying mechanism may be the spatial repulsion generated by the high affinity and compression of the PEG chain (Cennamo et al., 2015).

In addition, Liu and his colleagues devised a novel, enzyme-free, and versatile SPR sensor for detecting microRNA-21 and the human HCC cell line SMMC-7721. Their SPR aptasensor achieved a significantly enhanced SPR response using multiple signal amplification strategies where the target triggered the formation of a DNA super sandwich structure, and numerous positively charged AgNPs were absorbed (Liu et al., 2017).

Although AFP, as a star marker, has currently been utilized in clinical settings for the detection of various cancers, including HCC, gastric cancer, and breast cancer (Sun et al., 2017; Zhan et al., 2022), to date, there is no SPR aptasensor for AFP.

### 2.6 Other optical aptasensors

#### 2.6.1 Electrochemiluminescence aptasensors

The ECL technique combines the advantages and merits of the luminescence assay with the merits of electrochemical analysis, which has attracted considerable attention in developing ultrasensitive biosensing platforms for biomarker detection (Han et al., 2020; Hiramoto et al., 2020). Wu et al. (2014) developed an ECL imaging strategy for the simultaneous detection of multiple cancer biomarkers, including AFP, based on a closed bipolar electrode array (Wu et al., 2015). In addition, Zhang et al. (2015) described an ECL method based on DNA aptamer–target recognition and T7 exonuclease-assisted cycling signal amplification for VEGF-165 detection. Consequently, the ECL-based aptasensor had a detection limit of 0.2 pM within a wide range of VEGF-165 concentrations of 1 pM–20 nM (Zhang et al., 2015).

#### 2.6.2 Resonance light scattering aptasensor

The resonance scattering spectral (RLS) method has also been applied in biomarker aptasensors due to its simplification and rapidness (Gong et al., 2020). Based on the RLS technique, Chen et al. (2018) designed an intelligent and label-free sensor for the detection of AFP and miRNA-122 simultaneously. In the present study, cDNA1, which was part of the AFP aptamer strand region, hybridized with cDNA2, which was part of the region completely complementary to miRNA-122, to form double-stranded DNA. When the target (AFP or miRNA-122) was present, the cDNA (cDNA1 or cDNA2) could bind to the target. Therefore, RLS intensity changed proportionally with the concentration of AFP or miRNA-122. The proposed biosensor had detection limits as low as 0.94 μg/L and 98 pM for AFP and miRNA-122, respectively (Chen et al., 2018).

#### 2.6.3 Liquid crystals aptasensors

Liquid crystal (LC) sensors achieve target detection by transducing and amplifying chemical and biological events into visible optical signals. In the LC sensor by Duong and Jang (2021), the aptamer was immobilized on the surface of substrates, and then the binding of the AFP to its aptamer disrupted the LC alignment to generate the detection signal. The quantification limit of AFP achieved was 12.62 pg/mL (Duong and Jang, 2021). Qi et al. (2020) designed a platform where the two aptamers for AFP captured targets synergistically to format sandwich complexes of apt1/protein/apt2, which induced the release of signal DNA into the aqueous solution (Qi et al., 2020a).

## 3 Conclusion and future perspectives

In recent years, the global incidence and mortality of HCC have been on the rise. This review summarizes the development of optical aptasensors for biomarkers in the surveillance and early diagnosis of HCC. Each aptasensor based on an optical analysis strategy has its unique characteristics. Fluorescent aptasensors, one of the most prevalent sensors, can detect a large number of targets, whether in solution or on the membrane. However, there is a stability challenge referred to as photobleaching, which can affect the long-term usability of the sensor. Colorimetric aptasensors represent the simplest sensing mode, as they translate invisible signals into megascopic color changes. Based on this advantage, the colorimetric method has the most potential for commercial application outside the laboratory. Furthermore, the main challenge of such a type of sensor is that they have high detection limits compared to other methods. Although, as the signal amplification strategies progress, the problem is being resolved. Another optical-based strategy, chemiluminescence, has been exploited widely in recent years due to high sensitivity, wide calibration ranges, and simple instrumentation. Other optical analysis methods have also been recommended, including SPR, SERS, electrochemiluminescence, and resonance light scattering.

Studies associated with HCC based on optical aptasensors are disproportionate. VEGF is a prognostic monitoring marker for HCC and also a biomarker of multiple cancers (Volk et al., 2007); therefore, the detection of VEGF has been well studied. In contrast, there is a lack of optical aptasensors for detecting several commonly used serum biomarkers for HCC surveillance, such as DCP, Gp-73, IGF-1, and even AFP-L3, which is slightly more sensitive than AFP for HCC diagnosis in a biomarker screening test (Shiraki et al., 1995). The possible reason is that no corresponding high specificity and high-affinity aptamers for such markers have been selected. Additionally, although SPR is a widely used and highly sensitive detection method, to date, there is still not an aptasensor-based SPR for the star marker AFP.

Despite the considerable advantages of aptamer sensors over traditional methods in clinical analysis, some major challenges still warrant further investigation. Notably, defining biomarkers for diagnosing early HCC with better specificity and sensitivity scores than existing ones remains a challenge. In addition, despite the considerable specificity of aptamers in experimental studies, the detection accuracy of aptasensors is associated with false negatives or false positives in practical applications under the influence of complex matrixes of the samples. Consequently, few optical aptasensors have been made commercially available compared to the expansive academic literature in the area. Furthermore, the high development cost limits commercialization. Although the cost of aptamers is much lower than that of antibodies, there are still some enzymatic reactions and nanomaterials involved in aptasensors that increase their costs. Therefore, researchers should commit to producing commercial biosensors, which could facilitate the establishment of a clear consensus on HCC diagnosis and enhance visible light application. Finally, in cases where clinical studies are temporarily unable to provide a specific diagnostic marker, identifying multiple biomarkers simultaneously can improve detection efficiency and minimize false diagnosis cases. This will require the development and integration of more highly specific aptamers into the sensor platforms, posing a challenge to the specificity of the aptamer.

In view of the above challenges, future opportunities, and research directions are as follows:

First, the time is right for robust biomarker validation. Specifically, comparisons between early HCC patients and high-risk individuals with cirrhosis or with non-alcoholic fatty liver disease, rather than any stage HCC and the healthy subjects, are required. Besides, standardized collection, processing, storage, and analysis methods need to be established to ensure the consistency of clinical application. Prediction models combining multiple biomarkers with other parameters (such as sex and age) will emerge as trends.

Next, valuable biomarkers will naturally attract researchers’ interest with regard to the screening of their specific adapters. The aptamer with good selectivity and specificity is one of the keys to the performance of the aptamer sensor.

With the continuous optimization of functionalized nanomaterials and the increasing synergy with aptamers in the sensing fields, aptasensors can solve the bottleneck issue in detecting biomolecules with low abundance and monitoring ultra-weak biological signals in samples with complex stroma. For instance, molecular-imprinted polymers (MIPs) can provide rebinding interactions and spatial structures complementary to the target (Zhang et al., 2019). Numerous recent studies have used the apt-MIP hybrid probe to improve aptamer stability and strengthen the specificity of the probe. Integrating MIP-aptamers may be ideal candidates for novel hybrid nanoprobes with high specificity and affinity compared to other multi-hybrid composites. In addition, single-atom nanozymes (Wu et al., 2020) that contain maximum atomic utilization features and exhibit a highly assembled enzyme-like structure and remarkable enzyme-like activity (Liu D. et al., 2019; Lyu et al., 2020) enable signal amplification strategy already holds great promise in biosensors and shows satisfactory sensitivity and selectivity in biosensing (Jiao et al., 2021). In summary, considering the versatility of sensing substrates, the expansion of functionalized nanomaterials, the development of lab-on-a-chip and 3D printed devices, as well as microfluid-based nano aptamer sensors will advance and offer novel perspectives on the challenges of aptasensors in point-of-care diagnostics.

With advances in clinical research, nanotechnology, and signal amplification methods, and the tireless efforts of researchers, great strides will be made with regard to optical aptasensors, enabling the accurate and early diagnosis of liver and other cancers, providing patients with earlier diagnosis and longer survival probability.
